# A new ingestion bioassay protocol for assessing pesticide toxicity to the adult Japanese orchard bee (*Osmia cornifrons*)

**DOI:** 10.1038/s41598-020-66118-2

**Published:** 2020-06-11

**Authors:** Ngoc T. Phan, Neelendra K. Joshi, Edwin G. Rajotte, Margarita M. López-Uribe, Fang Zhu, David J. Biddinger

**Affiliations:** 1 Department of Entomology, The Pennsylvania State University, University Park, PA USA; 2 Research Center for Tropical Bees and Beekeeping, Vietnam National University of Agriculture, Hanoi, Vietnam; 3 Department of Entomology and Plant Pathology, University of Arkansas, Fayetteville, AR USA; 4 Penn State Fruit Research and Extension Center, Biglerville, PA USA

**Keywords:** Toxicology, Environmental sciences, Zoology, Entomology

## Abstract

Adopting an Integrated Pest and Pollinator Management strategy requires an evaluation of pesticide risk for pollinator species. For non-Apid species, however, the standardized ingestion assays are difficult to implement. This hinders the consideration of non-Apid species in farm management strategies and government regulatory processes. We describe a new method for a mason bee, *Osmia cornifrons*, which is an important pollinator of apples and other fruit crops. Our approach overcomes high control mortality seen in other bioassay protocols and expands testing to include males as well as females. The new pesticide toxicity assessment protocol showed that (1) a group feeding method is optimum even though there is no trophallaxis, (2) males had better tolerance to pesticides although they are smaller, and (3) pesticides can cause additional mortality after the standard 48 h assessment time specified by European Food Safety Authority and U.S. Environmental Protection Agency.

## Introduction

The global decline of pollinator populations has led to the need to develop an Integrated Pest and Pollinator Management (IPPM) framework that balances optimal pest control while ensuring pollinator health in agroecosystems^1^. Among multiple biotic and abiotic factors leading to pollinator decline, a major threat bees face while pollinating crops is the exposure to various insecticides, fungicides, herbicides, and even some plant growth regulators, which are applied to crops via foliar sprays, soil applications or seed treatments^2–4^. There are two main routes of pesticide exposure for foraging bees, contact with sprayed plant parts and ingestion with pollen and nectar^2^. Pollen and nectar can acquire pesticides applied via foliar applications applied pre-boom that translocate systemically through the plant vascular system into the nectar and pollen when flowers open^5–7^. Some pesticides applied during bloom can lead to contact exposure of pollinating insects^8,9^.

Honey bees (*Apis mellifera*) and bumble bees (*Bombus* spp.), both Apids, are the two taxa most frequently tested in bioassays that assess the risk and toxicity of pesticides to bee pollinators. Moreover, only female bees have been tested because males of these species are less abundant and much less important pollinators. Standardized test methodologies for assessing pesticide toxicity through contact and ingestion have been developed for these social species, but these methods cannot be utilized on non-social bees because of differences in behavior and rearing requirements^4,10,11^. Bees comprise a group of over 20,000 species that widely vary in physiology, ecology and behavior — about 70% of all species are solitary. The need for more robust protocols for pesticide risk assessment of pollinators has led to a high demand for the development of bioassays on solitary bee species^1,12^.

The Japanese orchard bee, *Osmia cornifrons* Radoszkowski (Hymenoptera: Megachilidae), is a solitary mason bee that is now managed as an alternative pollinator for the tree fruit industry^13–15^. *Osmia cornifrons* has been used to pollinate fruit orchards in Japan since the 1930s and was introduced to the U.S. in the 1970s by the U.S. Department of Agriculture (USDA) for this purpose^13,16^. The bees emerge from tubular nests (either manmade tubes or natural cavities in trees) as adults ready to forage in early spring when honey bee colonies have not yet reached their optimum strength and population size to pollinate crops in temperate climates^17,18^. *Osmia cornifrons* are short-ranged pollinators with a typical foraging range of 40–60 m and tend to stay in the orchards rather than flying among different crops or non-crop hosts like honey bees^13,19,20^. *Osmia* spp. are relatively easy to manage; the adults fly for only a few weeks of the year and once the adult flight period is over, completed nest tubes with larvae developing on pollen provisions can be removed from the field and stored until the next pollination season with minimal labor or costs for specialized beekeeping equipment^21,22^.

Bioassays to assess contact toxicity of systemic pesticides on adult *Osmia* have been performed, but bees are also exposed to systemic pesticides through ingestion^12^. Various adult ingestion methods have been attempted on the related red mason bee, *O. bicornis* (L.), and the European orchard bee, *O. cornuta* (Latreille) (mainly adapted from methods for honeybees and bumble bees), including group feeding and individual feeding methods such as the film canister method, glass vial method, and flower method^23–27^. Several experiments were conducted based on the European Food Safety Authority (EFSA) guidance document on pollinator risk assessment of pesticides. However, the success rates for these methods for adult solitary bee testing are very low due to high mortality, even in control groups. Indeed, based on the summary presentation of the International Commission of Plant-Pollinator Relationships (ICP-PR) non-*Apis* workshop 2017 (Valencia, Spain), the mortality rate in control treatment groups exceeded 20%^28^. Another potential problem is the time of mortality assessment. Presently, the U.S. Environmental Protection Agency (EPA) and EFSA regulations require mortality assessments at 48 h and 96 h, respectively^10,11^, but longer time periods may be necessary to capture delayed mortality.

All of the acute toxicity bioassays on *Osmia* spp. that have been conducted to date, involving both topical treatments and ingestion, have been done only on the females^12,29–31^. We evaluated the susceptibility of both females and males, not only because both take part in pollination processes and are exposed to orchard chemicals, but also because unlike social species where only a few males are needed to produce thousands of female worker offspring, sex ratios approaching 1:1 are typical in solitary bees. Without males, unmated female *Osmia* bees will only produce female-sized sons^32,33^ reducing the number of bees available for pollination. Bodyweight differences between males and females also need to be considered. Smaller individuals, males in *Osmia* species, tend to be more severely affected than larger bees when exposed to the same amount of a toxic substance^34^.

In this study, we (1) developed a standard protocol for ingestion toxicity assessment for non-Apid bees, using *O. cornifrons* as an exemplar; (2) examined the toxicity to both males and females of common pesticides applied to commercial apple trees; and (3) determined the optimal time after pesticide exposure to evaluate toxicity for ingested pesticides.

## Results

### Development of ingestion bioassay protocol

#### Optimal feeding method

**Figure 1:**
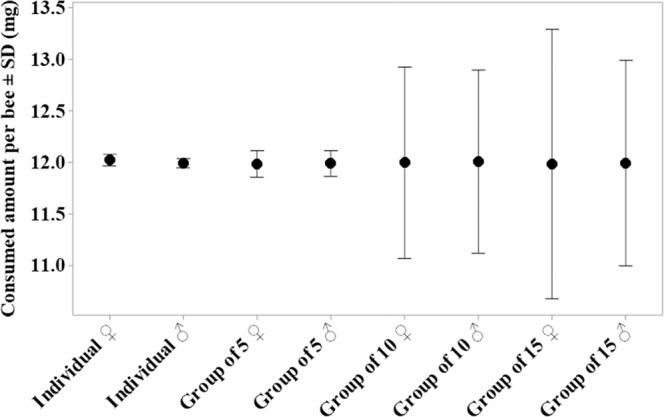
Consumed solution amount per adult *Osmia cornifrons* (in mg) for different feeding group sizes: individual feeding vs. group feeding (group of 5, group of 10, and group of 15 bees per container, respectively). Interval plot was created by Minitab 19; Dots show the mean and the error bars show standard deviations; Data table and further analysis can be found in the Supplementary Materials (Fig. S1 and Table S1).

The best method for this bioassay was feeding *O. cornifrons* in groups of five (Fig. 1). In this grouping, bees started feeding earlier and consumed all of the available food within the assigned four hours. This group feeding also allowed the even distribution of food (and dosing) among individuals during the assay (Levene’s test; Fig. S1 and Table S1). In contrast, when fed individually, bees took up to 48 hours to finish imbibing the solution. Moreover, bees tested individually delayed food ingestion; males delayed feeding for 12 hours and females for 24 hours (NP personal observation). For other group feeding methods (group sizes of 10 and 15 bees), there was obvious competition among the bees: some bees consumed greater amounts while the others consumed less than the average assigned food (10 uL of 50% W:W sucrose solution, which weighed 12 mg; Fig. S1 and Table S1).

#### Ingestion bioassay protocol

We repeated the *O. cornifrons* adult ingestion bioassay over two seasons (spring 2016 and spring 2017) using bees from the same source representing the same weight range (100–110 mg for females, 65–75 mg for males) and testing the same pesticide formulations (Table S2) in order to validate the group feeding method. A key point of this ingestion bioassay was to get the *O. cornifrons* to accept the feeders. We utilized two different feeding stimulation techniques. For the first year, we painted each feeding hole in the 2mL feeder tubes with a red Sharpie Permanent Marker. The red Sharpie was effective in stimulating the bees to feed. We do not know why it was a stimulus; whether it was a visual cue or an olfactory cue can be assessed in a future experiment. For the second year, we used smears of honey on the 2mL feeder tubes, which was better than the red Sharpie because it reduced handling time and also stimulated feeding. Instead of having to paint each feeder hole with the marker pen, we could easily use a honey-dampened Q-tip and swipe all of the feeder holes at once. In the second year (2017), we narrowed the pesticide concentrations to those more likely to cause 5–95% mortality in the test population, thus 95% CL of the LD_50_s (at 7 days after treatment) in 2017 were narrower than in 2016 (Table 1). The results of the two seasons were similar with 0% control mortality in both years indicating that our ingestion bioassay protocol for *O. cornifrons* was reproducible and the group feeding method was reliable for pesticide toxicity testing. The change in the stimulation feeding technique was an improvement and did not affect the final results.

**Table 1:** Comparison of toxicity responses of adult *Osmia cornifrons* to various pesticides through ingestion bioassays (at 7 days after treatment) conducted over two years (2016, 2017).

Active ingredient^1^	Sex	Year	N^‡^	LD_50_ (ng AI/mg bodyweight) (95% CL)	LD_50_ ratio (95% limits)
Thiamethoxam	♀	2016	225	0.034 (0.005–0.059)	1.11 (0.35–1.17)
		2017	225	0.030 (0.013–0.050)	
	♂	2016	225	0.011 (0.001–0.030)	1.55 (0.69–1.90)
		2017	225	0.007 (0.001–0.016)	
Imidacloprid	♀	2016	225	0.013 (0.009–0.028)	0.77 (0.58–1.45)
		2017	250	0.017 (0.016–0.019)	
	♂	2016	225	0.042 (0.019–0.057)	1.25 (0.65–1.52)
		2017	250	0.033 (0.029–0.038)	
Acetamiprid	♀	2016	225	0.14 (0.06–0.23)	1.25 (0.86–1.33)
		2017	270	0.11 (0.07–0.17)	
	♂	2016	225	0.55 (0.32–1.09)	0.93 (0.60–1.52)
		2017	270	0.59 (0.50–0.69)	

^1^The product formulations are listed in Table S2.

^‡^N is the number of individuals tested for each product. LD_50_ ratio in this table, or ratio of doses causing 50% mortality, is LD_50_ in 2016 ÷ LD_50_ in 2017. The bioassay result can be considered the same when the LD_50_ ratio is not significantly different from 1.0^46^. All 95% limits of LD_50_ ratios do overlap and include the value 1.0, indicating the response was similar in both years and validate the reproducibility of our protocol. Products are listed in decreasing order, from the most to the least toxic (based on quantal response bioassay results from POLOPlus 2.0).

### Sex-based differences in toxicity responses of *Osmia cornifrons* adults

Our data indicated over the two years that the females were more susceptible than the males for imidacloprid and acetamiprid (Table 2). No difference in response was recorded in the treatment containing thiamethoxam (Table 2). Again, the similarity of experiment results between the two years showed that our ingestion bioassay protocol was reliable.

**Table 2:** Sex-based differences in toxicity responses of *Osmia cornifrons* adults to various pesticides at 7 days after pesticide treatment.

Active ingredient^1^	Year	Sex	N^‡^	Sensitivity ratio (95% limits)
Thiamethoxam^§^	2016	♀	225	1.9(0.95–2.85)
		♂	225	
	2017	♀	225	2.38 (0.95–2.85)
		♂	225	
Imidacloprid	2016	♀	225	0.32* (0.47–0.51)
		♂	225	
	2017	♀	250	0.48* (0.32–0.67)
		♂	250	
Acetamiprid	2016	♀	225	0.19* (0.13–0.26)
		♂	225	
	2017	♀	270	0.20* (0.13–0.26)
		♂	270	

^1^The product formulations are listed in Table S2.

^‡^N is the number of individuals tested for each chemical. Sensitivity ratio, or lethal dose ratio at 50% mortality reading, is LD_50_ of female ÷ LD_50_ of male. Females are significantly (*) more susceptible than males to a product when the following requirements are met: (1) sensitivity ratio <1.0, and (2) 95% limit of sensitivity ratio does not include the value 1.0^46^. Otherwise, females and males can be considered to have the same response to a product. Control mortality was 0%. Products are listed in decreasing order, from the most to the least toxic (based on quantal response bioassay results from POLO Plus 2.0).

*Significant at the 95% confidence level.

^§^No difference in sex response.

### Delayed mortality assessment

Mortality of *O. cornifrons* varied over time in this study, requiring that each pesticide be evaluated at different intervals after exposure. Male mortality did not increase significantly at the LD_50_ level for imidacloprid after the 48 h mortality readings (Table 3). In contrast, thiamethoxam and acetamiprid continued to cause additional mortality in both sexes until the 7d assessment. Table 3 presents the mortality of *O. cornifrons* (2017) at different times post-treatment (2 days, 5 days, and 7 days) in different pesticide treatments. The result of 2016 was excluded because of its similarity with the result of 2017. It can be found in Supplementary Materials (Table S3).

**Table 3:** Toxicity response of *Osmia cornifrons* adults at different time intervals after pesticide treatment (2017).

Active ingredient^1^	Sex	N	Time of mortality reading^a^	Slope ± SE	LD_50_ (ng AI/mg bodyweight) (95% CL)	LD_50_ ratio (95% limits)
Thiamethoxam	♀	225	2	1.68 ± 0.20	0.077 (0.048–0.114)	
			5	1.53 ± 0.20	0.050 (0.025–0.078)	1.56* ^b^ (1.39–1.93)
			7	1.32 ± 0.19	0.030 (0.013–0.050)	2.53* ^c^ (2.28–3.60)
	♂	225	2	0.87 ± 0.18	0.016(0.01–0.04)	
			5	1.59 ± 0.43	0.009 (0.001–0.017)	1.90* ^b^ (1.74–1.98)
			7	1.66 ± 0.50	0.007 (0.001–0.016)	2.20* ^c^ (2.16–19.00)
Imidacloprid	♀	250	2	4.40 ± 0.59	0.023 (0.021–0.027)	
			5	4.16 ± 0.53	0.019 (0.017–0.021)	1.06* ^b^ (1.05–1.14)
			7	4.38 ± 0.53	0.017 (0.016–0.019)	1.16* ^c^ (1.12–1.27)
	♂	250	2	3.91 ± 0.54	0.033 (0.029–0.038)	
			5	3.76 ± 0.53	0.033 (0.029–0.038)	1.00 ^b^ (0.99–1.05)
			7	3.57 ± 0.52	0.033 (0.029–0.038)	1.00 ^c^ (0.99–1.01)
Acetamiprid	♀	270	2	0.94 ± 0.12	0.24 (0.15–0.35)	
			5	1.41 ± 0.15	0.13 (0.09–0.17)	1.78* ^b^ (1.71–2.20)
			7	1.61 ± 0.17	0.11 (0.07–0.17)	2.20* ^c^ (1.98–2.44)
	♂	270	2	2.93 ± 0.39	0.68 (0.55–0.83)	
			5	4.36 ± 0.80	0.59 (0.50–0.69)	1.09* ^b^ (1.03–1.15)
			7	4.36 ± 0.80	0.59 (0.50–0.69)	1.09* ^c^ (1.03–1.15)

^1^The product formulations are listed in Table S2.

Quantal response regression lines are represented by slope ± SE and LD_50_ (in ng AI/ mg bodyweight). LD_50_ ratio in this table, or ratio of lethal concentrations causing 50% mortality at different time reading (in this table, 2d vs 5d or 2d vs 7d), refers to the relative toxicity of a product at different time of reading compared with the standard 2d reading proposed by EPA or EFSA^4,10,11^. A product significantly continues to cause mortality after 48 h post-exposure when the following requirements are met: (1) LD_50_ ratio >1.0, and (2) 95% limit of LD_50_ ratio does not include the value 1.0^46^. Otherwise, delayed mortality can be considered to not occur. Most LD_50_ ratios were >1.0, indicating that most of the chemicals continued to cause mortality after 48 h. Products are listed in decreasing order, from the most to the least toxic (based on quantal response bioassay results from POLO Plus 2.0).

*Significant at the 95% confidence level.

^a^Mortality readings at 2, 5, and 7d after treatment.

^b^LD_50_ at 2d reading ÷ LD_50_ at 5d reading.

^c^LD_50_ at 2d reading ÷ LD_50_ at 7d reading.

## Discussion

We developed a robust and reproducible laboratory procedure to examine pesticide toxicity by ingestion in the solitary mason bee *O. cornifrons*. The consumption test showed that a cohort of five bees was the optimal number of bees per container for the ingestion bioassays even though *O. cornifrons* do not perform trophallaxis. Utilizing this procedure, toxicity responses of *O. cornifrons* to common orchard neonicotinoids revealed sex-differential responses, which showed that females are more susceptible than males (even when body mass was accounted for) for most of the pesticides examined. Since males in solitary bees are important for crop pollination^18,35^, pesticide impacts should be assessed for both sexes separately but females should have priority because of their higher susceptibility to pesticides. The reason for such sex-based differences in pesticide sensitivity or detoxification warrants further study^36^. Delayed mortality responses in *O. cornifrons* varied significantly across different pesticide active ingredients, with adult mortality reaching a maximum anywhere from 48 hours up to 7 days post-treatment. Presently, the EPA and EFSA regulations require assessment at 48 h and 96 h, respectively^10,11^. Based on our two-year study, we suggest that the mortality assessment protocols of these pesticide regulations should be revised.

Our results also contribute to the increasing evidence that the toxicity responses and detoxification mechanisms significantly vary across different bee families^12,37,38^. Future research should include comparing the susceptibility to pesticides to representatives of all bee families that significantly contribute to crop pollination including other members of the families Apidae, Megachilidae, Andrenidae and Halictidae. Pesticide risk assessments should not be based only on the responses of honey bees or bumble bees in cropping systems where significant pollination is being carried out by solitary bees^16,39–41^. We, and many others, question whether it is appropriate for pesticide policy to be made testing only honey bees that have vastly different behavioral, physiological and ecological traits compared to most other bee species^40^. Developing pesticide testing protocols for alternative pollinators is a step forward in understanding pesticide impacts on the majority of bee species.

We recommend group feeding in a cohort of five for *O. cornifrons* in future ingestion toxicity tests as this will ensure the bees consume the assigned amount within 4 hours of exposure. A series of pretests with different types of containers and different feeding stimulation techniques should be made to tailor the method for other species of solitary bees even within the same genus, as pre-trials with *O. lignaria* Say demonstrated much different feeding and foraging behaviors than *O. cornifrons* (NP and DB personal observation). We also recommend sex-based pesticide toxicity testing because male solitary bees also take part in pollination, are important for reproduction^35,42–44^, and can be the more-susceptible sex. Finally, we suggest that mortality assessment in pesticide toxicity tests should be extended beyond the current 48 hours to at least 7 days after treatment to ascertain the full mortality.

## Methods

### Collection and handling of the *Osmia cornifrons* for lab bioassays

*Osmia cornifrons* used for all bioassays were sourced from a single supplier (S. Famous, Harleysville, PA). Wild populations were trap nested in the supplier’s suburban backyard and from a nearby unsprayed park to minimize previous pesticide exposure prior to the bioassays. Both locations are at least 1,000 m from the nearest farm and isolated from agricultural pesticides. Binderboards (Pollinator Paradise, Parma, ID) type of mason bee trap nests, consisting of laminated wooden rearing blocks with 8mm-diameter holes lined with paper straws, were used. Adult emergence, pollen provisions, and egg-laying took place over several weeks starting in April and continuing through the first week of May in 2015 and 2016. Over the winter, straws containing the completed cocoons were stored in a screened insectary under ambient temperatures and protected from direct sunlight, rain, and snow.

In both years, the bees were received from the supplier in the winter as nest straws (extracted from nest tubes) containing adults in cocoons. The straws were processed, stored and prepared for release at the Penn State Fruit Research Center, Biglerville, PA as follows: The nest straws were peeled open to release the cocoons, which were then washed under running cold tap water to clean off kleptoparasitic pollen mites (*Chaetodactylus* sp., Acari: Chaetodactylidae), larval frass, pollen store remnants, and pieces of the mud partitions between cells. The damp cocoons were then air-dried at 20 °C for about 30 minutes on paper towels and stored at 2 °C to simulate winter temperatures until needed for experiments in February of 2016 and 2017. At that time, the overwintering, fully formed adults still in the cocoons were removed from refrigeration, then placed in 115mL plastic cups at 20 °C and allowed to emerge in insect cages (BugDorm-1 Insect Rearing Cage, MegaView Science Co. Ltd., Taiwan). To aid adult emergence from the cocoons, damp paper towels were lightly pressed onto the groups of loose cocoons in the plastic container to prevent movement of the cocoons which we found inhibited successful adult emergence, possibly due to lack of leverage for mandibles chewing through the tough cocoon. To further accelerate successful emergence, especially when large numbers of bees were needed to initiate an experiment, a small hole was made in the cocoon near the bee’s head using a dissecting needle.

**Figure 2:**
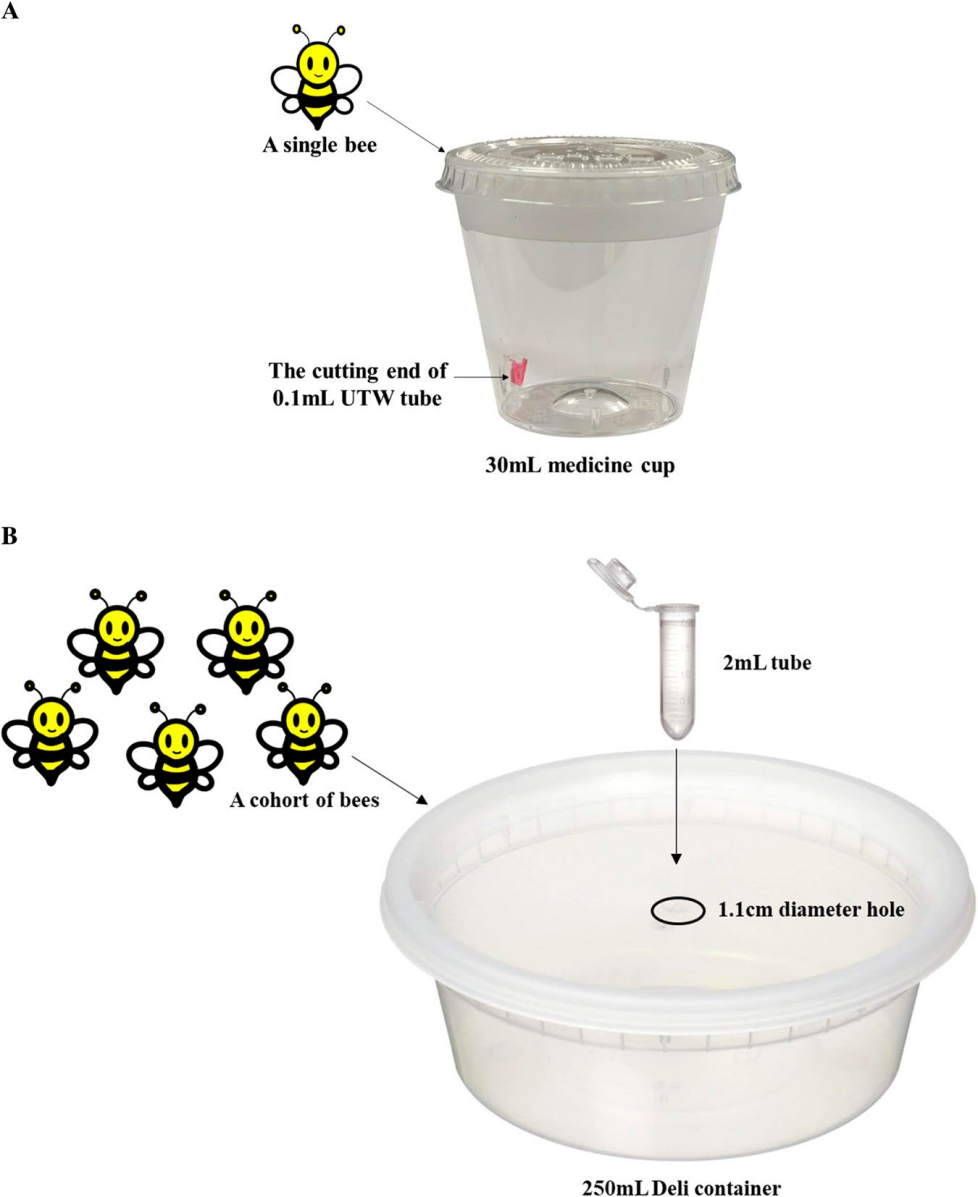
Feeding container design for *Osmia cornifrons*. (**A**) Individual feeding container*;* (**B**) Group feeding container.

In a separate preliminary step to minimize variation in testing, 50 one-day-old adult bees of each sex were randomly selected from the cages and sorted by body weight. For all bioassays, we used females that represented weights between 100–110 mg and males that represented weights between 65–75 mg. These weights approximate the mean of the weight distributions in the bee cohort (Fig. S2). *Osmia* adults were then caged into 250mL plastic containers for pesticide toxicity assessment. Each container had five same-sex individuals, males and females were kept separately to measure sex differences (Fig. 2B). Additionally, we noted that if both sexes were caged in the same container, mating activities would take priority over feeding, which delayed the feeding process and affected the final results of the bioassay (NP and DB personal observation).

### Test of consumption per bee

In order to determine whether each bee consumed the same amount of food, we firstly carried out a consumption test by feeding bees individually and in groups of 5, 10, and 15 bees per container (Fig. 1). We chose 165 one-day-old bees of each sex that were within the selected weight range (100–110 mg for females, 65–75 mg for males). The feeding solution was 50% W:W sucrose solution. Bee weight was assessed before being caged and after consuming the assigned solution. The consumed amount per bee (in mg) was the difference in a bee’s weight before and after feeding, given that *O. cornifrons* did not defecate in the feeding container (NP personal observation).

#### Individual feeding method

For the individual feeding method, each bee was kept separately in a 30mL plastic medicine cup with lid (Walgreens Co., Deerfield, IL). The feeder was made by cutting the bottom of a 0.1mL UTW tube (ThermoFisher Scientific Inc., Waltham, MA), then attaching it to the corner of the container with a glue gun (Fig. 2A). Ten microliters of sugar water were delivered to each bee by pipette. We then placed three of these containers into a 500mL plastic ‘Deli’ container (Harvest Pack Gourmet Showcase, Minneapolis, MN) to keep the bees calm.

#### Group feeding method

For the group feeding method, *O. cornifrons* received treatments inside 250mL plastic ‘Deli’ containers (Harvest Pack Gourmet Showcase, Minneapolis, MN). All sides of the containers were punched with 20 1mm-diameter holes to allow airflow. A small hole (1.2 cm diameter) was drilled in the lid to allow the introduction of live bees and extraction of dead bees. This hole was also used to attach a hanging feeder from the top. The feeder was a 2mL clear polypropylene graduated centrifuge tube with snap cap and round bottom (Globe Scientific Inc., Mahwah, NJ) with several pin-sized holes at the side near the bottom to allow *ad libitum* feeding (Fig. 2B). Using this method, we evaluated a cohort of 5, 10, or 15 bees per container. All bees in group feeding were marked by number tags (Betterbee, Greenwich, NY) and their weights before and after feeding were recorded. We provided 50, 100, or 150 uL sugar water to each group, respectively, in order to ensure bees received an average of 10 uL.

### Ingestion bioassay protocol

Candidate pesticide concentrations were mixed with 50% W:W sucrose solution. At first, we dissolved the pesticides in water, and then we diluted this solution with sucrose solution to get the desired concentrations. Table S2 lists the insecticides used in this study. We selected the range of treatment concentrations based on a previously determined field-relevant concentration. Each pesticide was tested at 1×, 3×, 10×, 30×, 100×, etc., of the mean of the concentration found in nectar and pollen from previous work^45^. These field-relevant concentrations were the pesticide levels in apple nectar and pollen measured after typical field application rates applied using a commercial air-blast orchard sprayer at 1 m^3^ of water/hectare at the pink growth stage (approximately five days before bloom for the pesticide-resistant Rosy Apple Aphid)^45^. In order to generate a dose-mortality curve, we chose 5–6 different doses (each with three replicates of 15 bees/replicate) that caused 5–95% mortality (after Robertson 2007)^46^.

For the ingestion bioassay, we maintained the bees, as described above for the consumption test, in groups of five bees per container. After 4 hours of starvation, the assigned treatment solution was fed to the group from the top of the containers. Bees were observed until they completely consumed the treatment solutions, then the treatment feeders were replaced with *ad libitum* 50% W:W sucrose solution. Three replicates of bees (each replicate contained 3 groups of five) were tested for each treatment concentration, for a total of 45 bees per dose. Containers were kept at 25 °C, RH 70%, with a photoperiod of 14:10 (L:D).

The plastic containers used to cage *O. cornifrons* were translucent to keep the bees calm. In clear containers, adult bees became agitated which affected their feeding (NP personal observation). In order to get the *Osmia* to accept the feeders, we either painted the feeder holes using a red Sharpie Permanent Marker (Newell Office Brands, NJ) (in 2016) or smears of honey (in 2017). In addition, we observed that the bees needed to defecate before they would feed, so they were left in the cages until they had defecated, then moved to the plastic containers.

### Assessment of sex-based response to pesticides

From the results of the bee consumption experiment, to increase the probability of equal food consumption per bee for *O. cornifrons*, we separated five bees of the same sex into one group, and three groups of five bees were considered as one replication. All treatment and control solutions were completely consumed by the bees within 4 hours.

### Delayed mortality assessment

In all experiments, mortality, which included dead and moribund bees, was recorded post-treatment at 12h intervals for the first 48 hours and every 24h after that until all bees were dead or 10 days had passed (after Biddinger 1998)^47^. Observed bees were divided into four main states: unaffected (no observable effects), weak (moving slowly); moribund (unable to move by themselves, trembling legs), and dead. Most of the weak bees recovered and moribund bees died subsequently. Bees were considered dead when they remained still during 30s of observation^48^.

### Statistical analysis

Quantal response regressions were conducted based on the response variables (states of bees) and explanatory variables (pesticide concentrations) with POLOPlus 2.0 (LeOra Software 2005) as described by Robertson 2007^46^. Regression lines represented the relationship between the response of bees and the concentration of pesticides, which allowed us to compare the toxicities of different pesticides by associating the concentrations that caused the same levels of mortality. LC_50_ (the concentration that kills 50% of the test population) values with 95% CI were calculated by POLOPlus 2.0 using the probit model. The lethal dose (LD_50_) of each pesticide to female and male *O. cornifrons* was calculated using the following formula:

$$L{D}_{50}\,({\rm{in}}\,{\rm{ng}}\,{\rm{AI}}/{\rm{mg}}\,{\rm{bodyweight}})=\frac{L{C}_{50}\,({\rm{in}}\,{\rm{ng}}\,{\rm{AI}}/{\rm{uL}}\,{\rm{dilution}})\ast 10{\rm{uL}}\,{\rm{dilution}}}{Average\,body\,weight(in\,mg)}$$

## Supplementary information

Supplementary Information.

## Data Availability

The datasets generated during the current study are available from the corresponding author on reasonable request.
