# Supplementary material for: A new ingestion bioassay protocol for assessing pesticide toxicity to the adult Japanese orchard bee (Osmia cornifrons)
Source: Sci Rep. 2020 Jun 11;10:9517. doi: 10.1038/s41598-020-66118-2 (PMC7289847; doi:10.1038/s41598-020-66118-2)
Supplement: Supplementary file 1 — Supplementary Information. [file 41598_2020_66118_MOESM1_ESM.docx]

**Supplementary Figures and Tables**

| **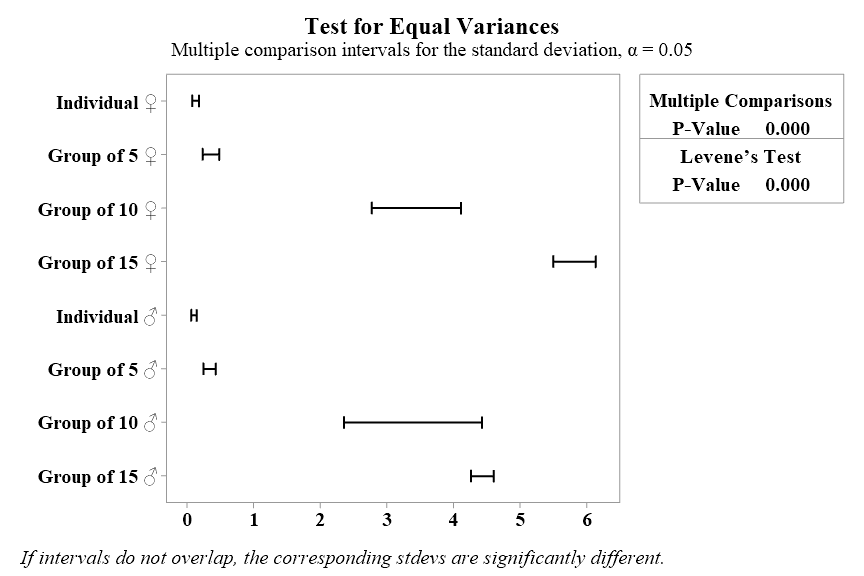** |
| --- |
| **Fig. S1: Test for homogeneity of variance among individual feeding and group feeding for adult *Osmia cornifrons*.** |
| All analyses were completed using Minitab 19;  Data table can be found in Table S1. |

| **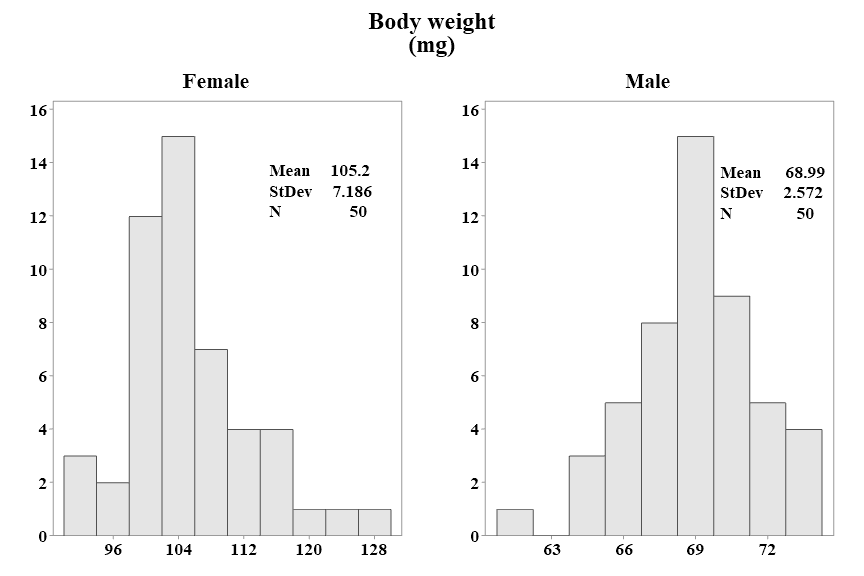** |
| --- |
| **Fig. S2: Weight distribution of 50 randomly selected *Osmia cornifrons* females and males.** |
| Histogram and analyses were completed using Minitab 19;  Bees were selected randomly from the same cohort. |

| **Table S1: Test for homogeneity of variance among individual feeding and group feeding** |
| --- |
| \| 95% Bonferroni Confidence Intervals for Standard Deviations \| \| \| \| \| --- \| --- \| --- \| --- \| \| **Sample** \| **N** \| **StDev** \| **CI** \| \| Individual ♀ \| 15 \| 0.10 \| (0.06, 0.21) \| \| Group of 5 ♀ \| 25 \| 0.31 \| (0.21, 0.50) \| \| Group of 10 ♀ \| 50 \| 3.27 \| (2.69, 4.20) \| \| Group of 15 ♀ \| 75 \| 5.69 \| (5.09, 6.58) \| \| Individual ♂ \| 15 \| 0.08 \| (0.05, 0.20) \| \| Group of 5 ♂ \| 25 \| 0.30 \| (0.20, 0.42) \| \| Group of 10 ♂ \| 50 \| 3.13 \| (2.38, 4.34) \| \| Group of 15 ♂ \| 75 \| 4.33 \| (3.91, 4.99) \| \| *Individual confidence level = 99.375%* \| \| \|  \| |

| **Table S2:** **Pesticides used in bioassays.**  The commercial name, common name and mode of action group for each pesticide are given. |
| --- |
| \| **Product** \| **Formulation** \| **Chemical Group**  **(IRAC Code)*** \| \| --- \| --- \| --- \| \| \| Actara 25WDG  *(Syngenta Crop Protection LLC,*  *Greensboro, NC)* \| Thiamethoxam 25% \| 4A – neonicotinoids \| \| Assail 30SG  *(United Phosphorus, Inc.,*  *King of Prussia, PA)* \| Acetamiprid 30% \| 4A – neonicotinoids \| \| Admire Pro 4.6F  *(Bayer CropScience LP,*  *Research Triangle Park, NC)* \| Imidacloprid 42.8% \| 4A – neonicotinoids \| |
| *IRAC: Insecticide Resistance Action Committee |

| **Table S3: Toxicity response of *Osmia cornifrons* adults at different time intervals after pesticide treatment (2016).** |
| --- |
| \| **Active ingredient**^1^ \| **Sex** \| **N** \| **Time of**  **mortality reading ^a^** \| **LD_50_ (ng AI/mg bodyweight)**  **(95% CL)** \| **LD_50_ ratio (95% limits)** \| \| --- \| --- \| --- \| --- \| --- \| --- \| \| Thiamethoxam \| ♀ \| 225 \| 2 \| 0.091  (0.032 – 0.149) \|  \| \| 5 \| 0.055  (0.012 – 0.098) \| 1.65* ^b^  (1.60 – 2.53) \| \| 7 \| 0.034  (0.005 – 0.059) \| 2.68* ^c^  (2.65 – 6.08) \| \| ♂ \| 225 \| 2 \| 0.042  (0.024 – 0.061) \|  \| \| 5 \| 0.025  (0.005 – 0.050) \| 1.68* ^b^  (1.28 – 4.56) \| \| 7 \| 0.011  (0.001 – 0.030) \| 3.82* ^c^  (2.14 – 22.80) \| \| Imidacloprid \| ♀ \| 225 \| 2 \| 0.049  (0.020 – 0.077) \|  \| \| 5 \| 0.035  (0.016 – 0.054) \| 1.40* ^b^  (1.19 – 1.50) \| \| 7 \| 0.013  (0.009 – 0.028) \| 2.89* ^c^  (2.11 – 3.77) \| \| ♂ \| 225 \| 2 \| 0.049  (0.024 – 0.075) \|  \| \| 5 \| 0.045  (0.023 – 0.066) \| 1.09* ^b^  (1.00 – 1.19) \| \| 7 \| 0.042  (0.019 – 0.057) \| 1.20* ^c^  (1.17 – 1.38) \| \| Acetamiprid \| ♀ \| 225 \| 2 \| 0.29  (0.16 – 0.42) \|  \| \| 5 \| 0.14  (0.07 – 0.23) \| 2.07* ^b^  (1.92 – 2.17) \| \| 7 \| 0.14  (0.06 – 0.23) \| 2.07* ^c^  (1.92 – 2.53) \| \| ♂ \| 225 \| 2 \| 0.83  (0.43 – 1.22) \|  \| \| 5 \| 0.68  (0.35 – 1.14) \| 1.17* ^b^  (1.12 – 1.22) \| \| 7 \| 0.55  (0.32 – 1.09) \| 1.28* ^c^  (1.18 – 1.51) \| |
| ^1^The product formulations are listed in Table S2.  LD_50_ ratio in this table, or ratio of lethal concentrations causing 50% mortality at different time reading (in this table, 2d vs 5d or 2d vs 7d), refers to the relative toxicity of a product at different time of reading compared with the standard 2d reading proposed by EPA or EFSA^4,10,11^. A product significantly (*) continues to cause mortality after 48h post-exposure when the following requirements are met: (1) LD_50_ ratio > 1.0, and (2) 95% limit of LD_50_ ratio does not include the value 1.0^46^. Otherwise, delayed mortality can be considered to not have occurred. Most LD_50_ ratios were >1.0, indicating that most of the chemicals continued to cause mortality after 48h of exposure. Products are listed in decreasing order, from the most to the least toxic (based on quantal response bioassay results from POLO Plus 2.0).  ***:** significant at the 95% confidence level  ^a^: mortality readings at 2, 5, and 7d after treatment  ^b^: LD_50_ at 2d reading ÷ LD_50_ at 5d reading  ^c^: LD_50_ at 2d reading ÷ LD_50_ at 7d reading |
